# Sister species diverge in modality‐specific courtship signal form and function

**DOI:** 10.1002/ece3.7089

**Published:** 2020-12-30

**Authors:** Eileen A. Hebets, Mitch Bern, Rowan H. McGinley, Andy Roberts, Arik Kershenbaum, James Starrett, Jason E. Bond

**Affiliations:** ^1^ University of Nebraska‐Lincoln Lincoln NE USA; ^2^ The Ohio State University at Newark Campus Newark OH USA; ^3^ University of Cambridge Cambridge UK; ^4^ University of California Davis Davis CA USA

**Keywords:** competition, condition dependence, multimodal, niche partitioning, sexual selection, signal space, speciation

## Abstract

Understanding the relative importance of different sources of selection (e.g., the environment, social/sexual selection) on the divergence or convergence of reproductive communication can shed light on the origin, maintenance, or even disappearance of species boundaries. Using a multistep approach, we tested the hypothesis that two presumed sister species of wolf spider with overlapping ranges and microhabitat use, yet differing degrees of sexual dimorphism, have diverged in their reliance on modality‐specific courtship signaling. We predicted that male *Schizocosa crassipalpata* (no ornamentation) rely predominantly on diet‐dependent vibratory signaling for mating success. In contrast, we predicted that male *S. bilineata* (black foreleg brushes) rely on diet‐dependent visual signaling. We first tested and corroborated the sister‐species relationship between *S. crassipalpata* and *S. bilineata* using phylogenomic scale data. Next, we tested for species‐specific, diet‐dependent vibratory and visual signaling by manipulating subadult diet and subsequently quantifying adult morphology and mature male courtship signals. As predicted, vibratory signal form was diet‐dependent in *S. crassipalpata,* while visual ornamentation (brush area) was diet‐dependent in *S. bilineata*. We then compared the species‐specific reliance on vibratory and visual signaling by recording mating across artificially manipulated signaling environments (presence/absence of each modality in a 2 × 2 full factorial design). In accordance with our diet dependence results for *S. crassipalpata,* the presence of vibratory signaling was important for mating success. In contrast, the light and vibratory environment interacted to influence mating success in *S. bilineata,* with vibratory signaling being important only in the absence of light. We found no differences in overall activity patterns. Given that these species overlap in much of their range and microhabitat use, we suggest that competition for signaling space may have led to the divergence and differential use of sensory modalities between these sister species.

## Graphical abstract

Using phylogenomic scale data, this study first confirms the sister‐species relationship of two wolf spiders—*Schizocosa crassipalpata* and *Schizocosa bilineata*—that overlap in range and habitat. Next, using a combination of diet manipulations and laboratory‐based behavioral assays, we demonstrate that the species diverge in the form and function of their courtship signaling. *Schizocosa crassipalpata* shows diet‐dependent vibratory signals, while visual ornamentation was diet‐dependent in *S. bilineata*. Coincident with this, vibratory signaling was important for mating success in *S. crassipalpata*, while there was an interaction between vibratory signaling and visual signaling for *S. bilineata*. We suggest that competition for signaling space has influenced signaling divergence in these sister species.

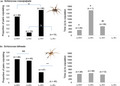

## 1 INTRODUCTION

Among closely related animal taxa, there often exist elaboration, complexity, and diversity in traits associated with signaling. Multiple birds, for example, express varied multimodal displays incorporating both song signaling and visual signaling (Gomes et al., 2017; Mason et al., 2014; Matysiokova et al., 2017; Ornelas et al., 2009), as do spiders (Elias et al., 2013; Hebets et al., 2013; Masta & Maddison, 2002) and frogs (reviewed in Starnberger et al., 2014). Even within individual signaling modalities, many closely related taxa express remarkable differences in signaling. We see, for example, great variation among electric signals of mormyrid fish (Arnegard et al., 2010; Hopkins, 1981). Visual displays of many birds (e.g., birds of paradise, Scholes, 2008, and manakins, Prum, 1990), visually based coloration or head‐bob displays in lizards (Chen et al., 2012; Martins et al., 2004), and color patterns in darters (fish) (Gumm & Mendelson, 2011) all provide examples of visual signal diversification. Understanding the origin and maintenance of such signal diversity remains at the heart of many evolutionary studies. Research testing the relative roles and importance of different drivers of signal divergence—for example, ecological, genetic, and social/sexual selection—remains an active and stimulating field of study (Bailey et al., 2019; Derryberry et al., 2018; Garcia et al., 2020; Hebets et al., 2013; Wilkins et al., 2018).

The environment can play a large role in signal evolution and function. Early studies on bird song demonstrated that different environments, or habitats, select for songs with different acoustic form (Hunter & Krebs, 1979; Morton, 1975). For visual signaling, the light environment influences the visual contrast of manakin displays, and individuals will alter their display location in order to increase visual contrast (Endler & Thery, 1996; Thery, 1987, 1990). The vibratory courtship displays of *Schizocosa* wolf spiders vary in their attenuation across different substrate types (Choi et al., 2019; Elias et al., 2010; Hebets et al., 2008), making vibratory signaling more or less effective across different microhabitats. Furthermore, communication systems have evolved in environments that influence not only signal transmission efficacy, but also the sensory and neural systems of receivers (Cummings & Endler, 2018; Endler, 1992). Environmental differences can be drivers of speciation (Boughman, 2002), but signal divergence is also observed in closely related animals that appear to share habitats.

In multispecies assemblages with overlapping habitat use, competition for effective communication is hypothesized to lead to species‐specific signal space, or divergent signaling among sympatric species. Numerous studies have tested, and found support for, this hypothesis in acoustic signaling animals (e.g., green lacewings: Henry & Wells, 2010, frogs: Sinsch et al., 2012, Chek et al., 2003, crickets: Schmidt et al., 2013). In addition to evolutionary divergence in signal form, signalers can also alter their signaling location, or timing, to overcome competition for signal space, demonstrating plasticity in signaling behavior. Four sympatric species of wren warbler (Cisticolidae, *Prinia*), for example, partition acoustic signal space and song perch heights, presumably to overcome heterospecific signal interference (Chitnis et al., 2020). The extent to which competition for signaling space per se influences the evolution of novel signaling traits or signaling modalities in sympatric populations, however, remains an open question.

This study tests the hypothesis that two proposed sister species of *Schizocosa* wolf spider—*S. crassipalpata* and *S. bilineata* (Stratton, 2005; Vaccaro et al., 2010)—that differ in their degree of sexual dimorphism, yet overlap in habitat use, have diverged in their modality‐specific signaling. In particular, we propose that secondary sexual traits involved in visual courtship signaling, as well as an increased reliance on visual signaling (as opposed to vibratory) for mating success, have evolved in *S. bilineata*.

*Schizocosa* wolf spiders are non‐web‐building, ground‐dwelling spiders common to a variety of habitat types across North America (e.g., grassy fields, deciduous forests, pine forests) (Dondale & Redner, 1978; Stratton, 1991, 1997, 2005). While species within this genus may overlap generally in their ranges (Dondale & Redner, 1978), they are often found in association with specific microhabitat characteristics, such as substrate type (e.g., Rosenthal et al., 2019). The choices of microhabitat use (substrate type) and the properties of substrate‐specific vibratory signal transmission are important to the mating success of multiple *Schizocosa* species (*S. floridana*: Rosenthal et al., 2019, *S. retrorsa*: Hebets, Elias, et al., 2008, *S. stridulans*: Elias et al., 2010, *S. retrorsa*: Choi et al., 2019). In fact, differential microhabitat use is suggested to have been an important factor leading to the divergent morphologies and courtship displays observed in the closely related species *S. ocreata* and *S. rovneri* (Stratton, 2005; Stratton & Uetz, 1981, 1986; Uetz & Denterlein, 1979). Moreover, in a mixed population of genetically indistinguishable ornamented and unornamented *Schizocosa* (Hebets & Vink, 2007), a combination of allochrony, microhabitat use, and mate choice is proposed to be driving speciation (Fowler‐Finn et al., 2015; Gilman et al., 2018).

Coincident with the production of vibratory courtship songs, many *Schizocosa* species move and wave ornamented forelegs (possessing dark pigmentation and/or black brushes) as part of presumed visual courtship displays (Dondale & Redner, 1978; Hebets et al., 2013; Stratton, 1991, 1997, 2005). Previous studies on select species have found the diet‐dependent expression of male foreleg ornaments (*S. ocreata*, Uetz et al., 2002, *S. uetzi*, Shamble et al., 2009, *S. floridana*, Rosenthal & Hebets, 2012, *S. stridulans*, Rosenthal & Hebets, 2015), suggesting sexual selection for these traits. However, even ornamented species appear to rely predominantly on vibratory signals for mating success (reviewed in Hebets et al., 2013). The documented variation in multimodal signaling form and function across the North American genus *Schizocosa* makes this group an especially attractive system for studying the evolution and diversification of courtship signaling.

There is one *Schizocosa* species pair that differs in their degree of sexual dimorphism, yet overlaps in their range, their apparent microhabitat use, and their seasonality. In North America, *S. crassipalpata* and *S. bilineata* range from southern Canada south to the U.S. states of Georgia and Texas, and from the East Coast to as far west as South Dakota, Nebraska, and Kansas (Comstock, 1940; Dondale & Redner, 1990; Guarisco, 2020; Kaston, 1948; Sierwald et al., 2005; Stratton, 2005; Vaccaro et al., 2010). Their distributions largely overlap and one can find both sympatric and allopatric populations (M. Bern & EA Hebets, personal observation). The two species are often noted as resembling one another, except for the black tibial brush on mature male *S. bilineata* (Dondale & Redner, 1978). Both species are spring‐active, and both can be found in grassy fields, meadows, and short, mixed, and tall grass prairies (among others) (Dondale & Redner, 1978; Guarisco, 2020). The sexual behavior of *S. bilineata* has previously been described (Kaston, 1936; Montgomery, 1903; Vaccaro et al., 2010), but the condition dependence or importance of vibratory and visual courtship signaling has yet to be explored in either species.

Phylogenetic analyses using morphological and mitochondrial data previously hypothesized that *S. crassipalpata* and *S. bilineata* are sister species (Fowler‐Finn et al., 2015; Stratton, 2005). If confirmed, these two species represent an example of closely related taxa with *conspicuously* divergent courtship signaling—a monomorphic species with an asynchronous vibratory and visual display and a sexually dimorphic species with visually prominent ornamentation (foreleg brushes) and synchronized vibratory and visual signaling (see *Objective I*—*Focal Species Morphology and Behavior* for courtship details). Through a series of studies, we test the hypothesis that *S. crassipalpata* and *S. bilineata* have diverged in the sensory modalities that they rely upon for courtship signaling. We first used molecular data to rigorously test the previously hypothesized sister‐species relationship (proposed based upon morphological, Stratton, 2005; and mitochondrial data, Fowler‐Finn et al., 2015) between *S. crassipalpata* and *S. bilineata* (Objective I).

Next, we tested the diet dependence of vibratory and visual signaling in both species by artificially manipulating spider diets and comparing and contrasting the influence of diet manipulations (high versus low quantity) on vibratory and visual signal form (Objective II). We predicted vibratory signal form to be diet‐dependent in *S. crassipalpata* and visual signal form to be diet‐dependent in *S. bilineata*. We then directly tested the function of vibratory and visual signaling across both species by examining their relative roles in successful mating. We accomplished this by quantifying mating success of species pairs across signaling environments that varied in their capacity to transmit visual and/or vibratory signals (Objective III). Finally, we tested the hypothesis that *S. crassipalpata* and *S. bilineata* have diverged in activity patterns by quantifying the activity of females and males of both species during lights on versus lights off periods (Objective IV).

## 2 OBJECTIVE I—EVOLUTIONARY RELATIONSHIPS BETWEEN SPECIES

### 2.1 Focal species morphology and behavior

*Schizocosa crassipalpata* males lack foreleg ornamentation and their courtship consists of the production of a series of substrate‐borne vibrations (vibratory signaling) and an asymmetrical tapping of the unornamented forelegs (visual signaling) (Dondale & Redner, 1978; Stratton, 2005) (Video S1). The dynamic visual display is not synchronized with, but instead follows, the vibratory display.

In contrast to the unornamented *S. crassipalpata* males, *S. bilineata* males possess dark pigmentation and dark brushes on the tibia of their forelegs at maturation (Dondale & Redner, 1978; Stratton, 2005; Vaccaro et al., 2010). Similar to courting male *S. crassipalpata*, *S. bilineata* males produce a series of substrate‐borne vibrations during courtship. *Schizocosa bilineata* males also incorporate visual signaling characterized by an incremental lowering of one or both ornamented forelegs that culminates in a quick tap of the substrate (Vaccaro et al., 2010) (Video S2). In contrast to the asymmetrical multimodal signaling of *S. crassipalpata*, the vibratory and visual displays of *S. bilineata* occur in synchrony.

Previous studies (Fowler‐Finn et al., 2015; Hebets et al., 2013; Stratton, 2005) based on limited character sampling hypothesize a sister group relationship between these two species; we explicitly test that hypothesis using a genomic scale data set.

### 2.2 Methods

We generated DNA sequence data for *S. crassipalpata* and *S. bilineata*, seven other species of *Schizocosa* from North America and the lycosid genus *Varacosa,* which we used to root the tree (Table S1). We extracted total genomic DNA from leg tissue using the DNeasy Blood & Tissue Kit (Qiagen). We determined DNA concentration using a Qubit 2.0 (Thermo Fisher Scientific) and evaluated DNA quality using gel electrophoresis. Library preparation, enrichment, and sequencing were conducted at the Center for Anchored Phylogenomics at Florida State University (http://www.anchoredphylogeny.org) following (Hamilton et al., 2016; Lemmon et al., 2012; Maddison et al., 2017). Indexed samples were pooled at equal quantities (16 samples per pool), and then, each pool was enriched using the AHE Spider Probe Kit v1 (Hamilton et al., 2016) and a modified v2 kit developed for targeting araneomorph libraries (Maddison et al., 2017). Paired‐end 150 base pair reads were generated with Illumina HiSeq 2500 at the Florida State University Translational Science Laboratory in the College of Medicine. Read pair merging, reference assembly, orthology inference, and sequence alignment and trimming followed (Hamilton et al., 2016; Maddison et al., 2017). We performed a maximum‐likelihood (ML) phylogenetic analysis on the concatenated data set, partitioned by locus, using RAxML v.8.2.9 (Stamatakis, 2014) on the Auburn University Hopper cluster. Nodal support was determined with 1,000 random sequence additions and 1,000 bootstrap replicates; an independent GTR+G model was defined for each partition. We conducted additional ML analyses in IQ‐TREE (Nguyen et al., 2015). Model selection for each partition was performed with *ModelFinder Plus*; support values were inferred via ultrafast bootstrapping (1,000 replicates) and SH‐aLRT.

### 2.3 Results

**Figure 1:**
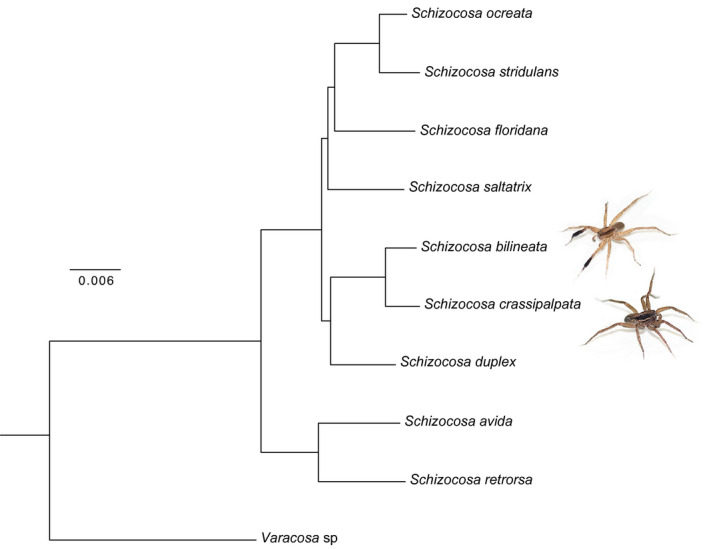
Phylogeny of *Schizocosa* showing the sister relationship of *Schizocosa crassipalpata* and *Schizocosa bilineata*. Tree topology is based on maximum likelihood, with branch lengths in units of substitutions per site. Bootstrap values at all nodes are 100 percent

We recovered 533 loci for a total concatenated alignment length of 302,711 nucleotides with 13.13% missing data. Sequence alignments and Illumina short reads are available in Dryad (doi: 10.25338/B8933F). The best tree resulted in a log likelihood of −607,873.05260 and −607,970.878 for the RAxML and IQ‐TREE analyses, respectively (Figure 1). Bootstrap and SH‐aLRT support values are 100 percent at all nodes. We recovered the sister relationship of *S. crassipalpata* and *S. bilineata*, consistent with phylogenies based on mitochondrial sequences and morphology (Fowler‐Finn et al., 2015; Hebets et al., 2013; Stratton, 2005). An expanded forthcoming analysis including all North American *Schizocosa* finds this same relationship (Starrett et al. in prep). Together, *S. crassipalpata* and *S. bilineata* are sister to *S. duplex*, a relationship that has not previously been reported.

## 3 OBJECTIVE II—DIET DEPENDENCE IN MULTIMODAL SIGNALING

The form of individual communication signals, or components, and their relationship with other signaler traits (e.g., signaler size, immunity, and age) is often used as a proxy of signal “function”—for example, as indicator traits (O’Steen et al., 2010; Ryan, 1980). In the context of courtship communication, for example, many signaling traits are sexually dimorphic, exaggerated, and are assumed to be under directional selection (e.g., Darwin, 1871; Delcourt & Rundle, 2011; Rosenthal & Evans, 1998). Furthermore, any benefit to sexual fitness associated with expressing such traits is often proposed to be offset by the cost of survival, making sexually selected signaling traits costly (Bonduriansky, 2007; Cotton et al., 2004b). Due to the presumed cost associated with exaggerated sexual signaling traits, theory predicts that they should develop a higher degree of condition dependence than nonsexually selected traits (Bonduriansky, 2007; Cotton et al., 2004b; Rowe & Houle, 1996). As such, testing the condition dependence of signaling traits (e.g., their form in relation to diet) can provide insight into their potential function.

### 3.1 Methods

#### 3.1.1 Collection and maintenance

We collected immature *S. crassipalpata* from an open grassy habitat (canopy height < 250 mm) in the Bath Nature Preserve in Summit County, OH (41.1766°N, 81.6480°W), from 17 to 21 March 2009. All *S. crassipalpata* individuals were 1–2 molts from maturity at collection. We collected immature *S. bilineata* in an open grassy habitat (canopy height < 200 mm) along a riparian zone near the north end of The Ohio State University campus at Newark, Licking County, OH (40.0753°N, 82.4425°W), from 29 to 31 March 2010. Though we collected each species at different sites, both species are at both sites—that is, they are sympatric in the populations from which they were collected. All *S. bilineata* individuals were 2–3 molts from maturity at collection. We collected immature spiders both to ensure that they were virgins and to allow for enough developmental time for diet manipulations to take effect (see below). After collection, we immediately transported all spiders back to the University of Nebraska‐Lincoln and housed them individually in 59 × 59 × 77 mm clear plastic containers (AMAC Plastic Products) with visual barriers between containers. We maintained all spiders on a 12‐hr:12‐hr light:dark cycle and provided them with a constant source of water.

#### 3.1.2 Experimental design

We manipulated the diet of subadult spiders to examine the effects of past diet on the expression of (a) adult male and female morphology and (b) vibratory and visual courtship signals. We randomly assigned subadult spiders of both species to one of two diet treatments: (a) low‐quantity diet (LD)—one cricket (*Acheta domesticus*, Bassetts cricket ranch, CA, USA) of a size visually approximate to the body of the spider (prosoma + opisthosoma) once per week, and (b) high‐quantity diet (HD)—one size‐matched cricket as above twice per week. We started the assigned feeding regimes immediately upon setup in the laboratory. Because immature individuals of both sexes are indistinguishable, we did not know the sex of individuals at the start of diet regimes. We examined all spiders at least every 2 days for molts to estimate the date of their final maturation molt. Once mature, we recorded each spider's mass.

#### 3.1.3 Diet and courtship signal form

Due to a lack of information on age‐specific courtship performance in either species, we attempted to control for male age across treatment groups. We made recordings on a vibration isolation table (Minus K 50BM‐8C; Minus K Technology) in a sound isolation chamber measuring 500 × 370 × 430 mm. The chamber was insulated with sound‐absorbing foam (Super Soundproofing Co.) and fitted with a Vita‐Lite full‐spectrum florescent bulb (Duro‐Test Lighting Inc.). The temperature in the testing chamber was 22°C (±1°). We used a Laser Doppler Vibrometer (Polytec PDV‐100; Polytec), set for a peak velocity measurement range of ±20 mm/s, with a low‐pass filter at 22 kHz, and at a 24 bit 48 kSa/a sample rate for all vibratory recordings. We recorded output from the vibrometer on an Apple PowerBook using a power 11401 A‐to‐D converter and Spike 2 version 5 (Cambridge Electronic Design Ltd). We exported all recordings as uncompressed WAV files for signal analysis.

We recorded vibrations from males residing in a testing arena set inside the sound isolation chamber on the vibration isolation table. We lined the bottom of the arena with filter paper containing conspecific female cues. Three high‐diet conspecific females deposited silk cues on the filter paper for two hours prior to trials. We cut up the impregnated filter paper into smaller pieces that we spread across the floor of the arena. In this way, we exposed each male to cues from all three females during the trials. Male *Schizocosa* have been shown to assess female reproductive status and receptivity based on silk cues (Roberts & Uetz, 2005), and thus, our design attempted to control for variability in female silk cues. Additionally, using three separate females allowed us to overcome potential issues associated with using only a single stimulus source. We used different sets of three females for each focal male.

We placed a 5 × 5 mm piece of retroreflective tape (3M Diamond Grade; 3M, Saint Paul, MN, USA) in the center of the filter paper to increase the signal strength of the vibrometer. We introduced males into the testing arena and allowed them to acclimate for up to ten minutes. If the male did not begin chemo‐exploration by the end of the acclimation period, we removed him from the arena and ended the trial. If a male did not begin to court in the first five minutes following chemo‐exploration, or did not produce more than a single courtship bout, then again, we removed the male and ended the trial. For males that did court, we recorded five minutes of courtship. If a male did not produce more than two bouts of courtship during the recording period, we discarded the recording and did not use it in our analysis.

**Figure 2:**
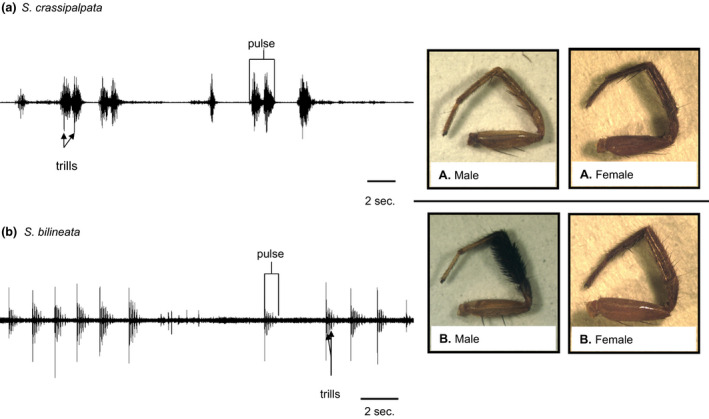
Vibratory signal form and foreleg ornamentation in (a) *Schizocosa crassipalpata* and (b) *Schizocosa bilineata*

Because we allowed males to move freely in the arenas during trials, we do not have information about a male's exact location relative to the laser while they were courting. It was thus impossible to accurately measure characteristics of signal amplitude from our recordings. Both species' signals were broadband in nature, and the signal characters initially scored for both species were number of pulses, number of trills (Figure 2, left panel), and total time spent courting (i.e., time from first courtship to end of recording). We quantified all signal characters using Raven Pro version 1.3 (Cornell Laboratory of Ornithology).

To quantify aspects of morphology that could impact visual courtship signaling, we sacrificed spiders using cold storage and then photographed them using a SPOT Flex 15.2 64 Mp camera (SPOT Imaging Solutions) mounted on a light microscope (Leica Microsystems) following experiments. Since these spiders only live one year, they were all near the end of their natural life.

We took two photographs of each individual—one of the cephalothorax (prosoma or first body part) and one of the exterior lateral sides of one of the first walking legs, or foreleg (Figure 2, right panels). Using these photographs, we recorded measurements of the cephalothorax width (the distance between the two widest parts of the cephalothorax—CW) and total foreleg length (femur + patella + tibia + metatarsus + tarsus). We measured these morphological traits using Image‐Pro Discovery software (Media Cybernetics). We included a quantification of foreleg length because both species use their forelegs in dynamic visual signaling during courtship. Additionally, we took measurements of total brush area for all photographs of *S. bilineata* male forelegs. For all measurements, we measured each individual three times and used the mean of the three measurements in our analyses. In addition to male measurements, we quantified cephalothorax width (CW), foreleg length, and mass for females of each species across diet as well. We did this to disentangle foraging effects on general morphology from its influence on signaling‐specific traits (Cotton et al., 2004a, 2004b, 2004c).

Using a different set of males than those used for the vibratory signal recordings (due to logistics of acquiring relevant information from individuals within a limited time window), we also made high‐speed video recordings of 23 *S. crassipalpata* (11 HD and 12 LD) and 22 *S. bilineata* (10 HD and 12 LD) courting males. Because we lack information on age‐specific courtship performance in either species, we again controlled for male age across treatment groups by restricting our age range for males to 10–37 days postmaturation molt.

We recorded high‐speed videos of courting males using a Fastcam 1024 PCI high‐speed digital camera (Photron USA) at 500 fps. We illuminated spiders from above with two Lumina fiber–optic lights (Chiu Technical Corp.) and filmed from the side in clear plastic arenas measuring 130 mm wide, 135 mm high, and 35 mm deep. We fastened a grid of 1 mm squares to the back wall of the arena so that we could track movements and analyze them in a 2‐dimensional plane (*x*‐ and *y*‐axes). The shallow depth of the arenas helped to facilitate this 2‐dimensional analysis by limiting the spiders' movement in the *z*‐axis. We again lined the bottom of the arena with filter paper upon which we placed silk cues from 3 conspecific high‐diet females. We placed males in the arena to acclimate for up to 10 min. As with the vibratory signal recordings, if a male did not begin to chemo‐explore within the 10‐min acclimation period or if he did not begin to court in the first five minutes after chemo‐exploration, then we removed him and ended the trial. For males that courted, we recorded and analyzed the first courtship bout.

We analyzed the videos of courtship using Pro‐Analyst motion tracking software (Itronx Imaging Technologies Inc.). We quantified the same leg wave characteristics for both species. Specifically, we measured the height (mm) of the highest leg wave and the duration of that leg wave. We chose these leg wave characters because motor movements that are near the anatomical and physiological production limits, such as higher and faster leg waves, may provide information about male quality to females (Byers et al., 2010). We determined the height of the leg wave by measuring the distance from the tip of the last leg segment (tarsus) to the substrate directly below it. We determined the duration of the wave by measuring the time it took the tip of the tarsus to reach the substrate from the highest point of the leg wave. Unfortunately, because these dynamic movement displays were recorded from a distinct set of individuals than our vibratory and morphological traits, we were unable to include them in our large combined analysis. In other words, we were unable to directly test relationships among vibratory and visual signal from within and across individuals (e.g., Rosenthal et al., 2018), as we used different individuals for quantifying the vibratory and visual traits.

*Vibratory Signal Analyses*—To test for a high degree of intercorrelation between our quantified vibratory signal characters, we carried out principal component analyses on the correlation matrices of both species. We included in our matrix the number of pulses, number of trills, and time spent courting. Since the raw vibratory signal characters all had positively skewed distributions, we log‐transformed them prior to the principal component analyses. The first principal component (PC1) for *S. crassipalpata* (*N* = 25) had an eigenvalue of 2.94 and accounted for 98.13% of the variation in vibratory signaling. The first principal component (PC1) for *S. bilineata* (*N* = 26) had an eigenvalue of 2.94 and accounted for 98.04% of the variation in vibratory signaling. In all subsequent analyses, we used PC1 for each species as our index for vibratory courtship signaling instead of using each signaling component. Again, for both species, PC1 included number of pulses, number of trills, and time spent courting.

To provide baseline, qualitative information on the potential for competition in vibratory signal space, we compared temporal components of *S. crassipalpata* and *S. bilineata* courtship, looking specifically at degree of overlap. To gain a broader picture of expected temporal characteristics of nonsister species, we additionally examined the vibratory signal characteristics of their closest relative—*S. duplex*.

**Figure 3:**
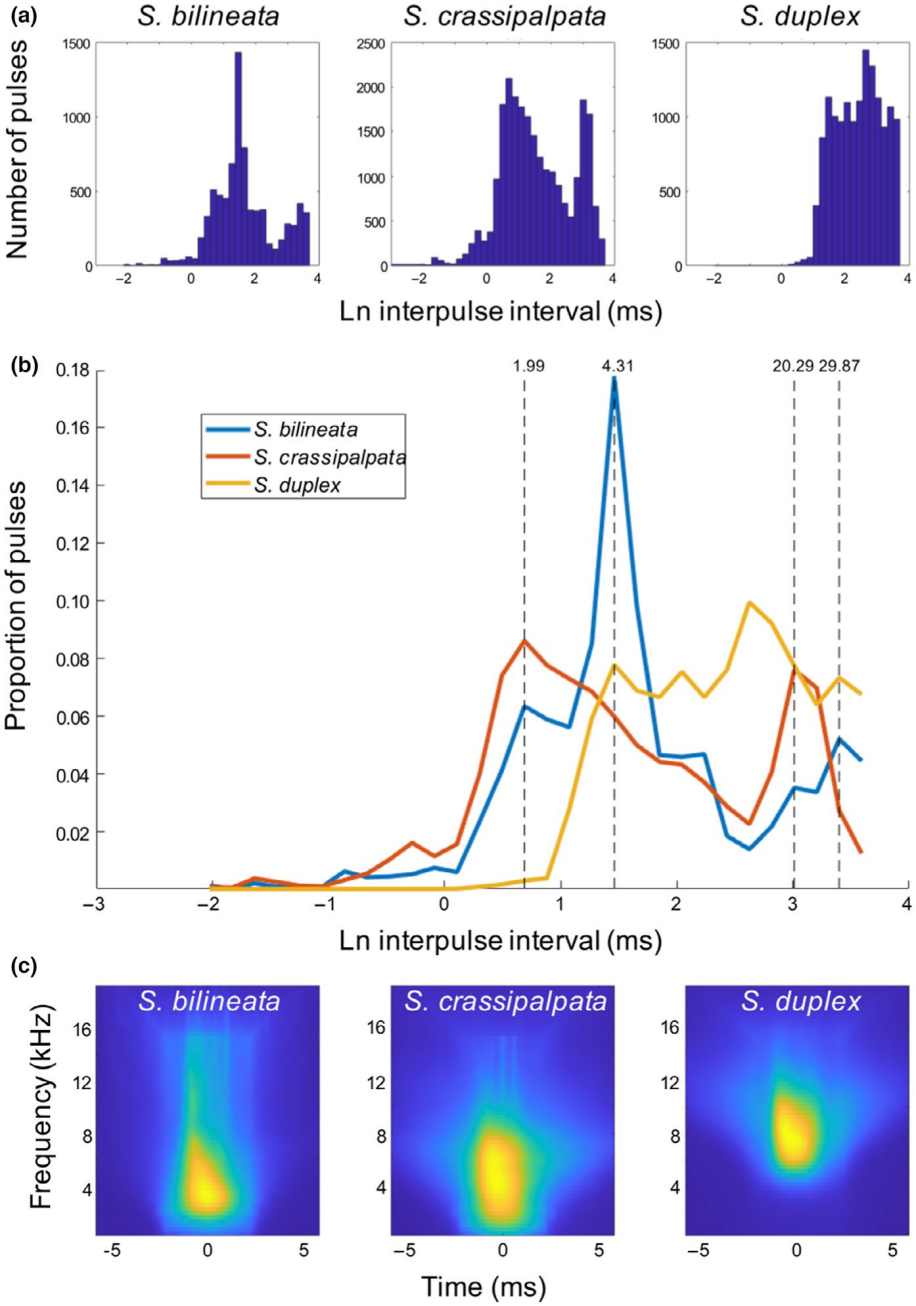
(a) Histograms of the log‐interpulse intervals for the three different species show a bimodal distribution of interpulse intervals in *Schizocosa bilineata* and *Schizocosa crassipalpata*, while *Schizocosa duplex* intervals are more uniformly further apart. (b) Histograms of the log‐interpulse intervals for the three species, overlaid on each other, indicate differences in interpulse intervals. Dashed lines represent the prominent peaks in incidence, with the interpulse interval in milliseconds indicated at the top. (c) Wavelet decomposition representation of the pulses for the three species show strong overlap. Each figure is an average composite of 10,000 individual pulses, generated using the Matlab *cwt* function, with analytical wavelet “*morse*.” *S. duplex* is dominated by high frequently wavelets more than *S. bilineata* and *S. crassipalpata*

We found that the interpulse interval of all three species show overlap, but with clear differences (Figure 3). *Schizocosa bilineata* and *S. crassipalpata* both show a bimodal distribution of interpulse intervals, with some pulses closer together (2–4 ms), and some further apart (20–30 ms) (Figure 3). *Schizocosa duplex* pulses are more uniformly further apart (>5 ms). The spectral structures of the pulses, as determined by performing a wavelet transformation (using the Matlab function *cwt* with analytical wavelet “*morse*”) in contrast, show broad similarities, although *S. duplex* pulses appear to be dominated by high‐frequency wavelets more than *S. bilineata* and *S. crassipalpata* (Figure 3).

*Visual Signal Analyses*—Because our visual signal measurements included morphological traits that were all highly correlated (e.g., body measurements such as cephalothorax width (CW) and leg length), we used residuals to examine the effect of diet on each trait. We used CW as our proxy for body size since this remains fixed at maturation. For each species and sex, we separately regressed leg length, mass, and brush area (for *S. bilineata* males only) against CW and calculated residuals for each. We used residual leg length as our measure of leg length independent of body size (hereafter “relative leg length”). We regressed residual mass against residual leg length to give an estimate of mass independent of body size and leg length (hereafter “relative mass”). For *S. bilineata* males, we added an extra step of regressing residual brush area against residual leg length and then against residual mass to give a measure of brush area independent of body size, leg length, and body mass (hereafter “relative brush area”). We then performed *t* tests to examine the effect of diet on each of these measures and the effects of diet on vibratory and visual courtship signaling. Due to the number of tests, we implemented a false discovery rate procedure (Benjamini & Hochberg, 1995) to adjust our threshold for determining statistical significance.

We used multiple linear regression to examine the effects of diet, morphological traits (i.e., CW, relative leg length, relative mass, and relative brush area—for *S. bilineata* only), and interactions between diet and morphological traits on vibratory (PC1) and dynamic visual (leg wave duration and relative wave height) courtship traits. Since diet influenced morphological traits, we standardized them by diet prior to including them in models with diet as a predictor variable. For model selection, we used the Akaike information criterion with a correction for small sample sizes (AICc). We ran all statistics using JMP v 9.0 (SAS Institute Inc.).

### 3.2 Results

#### 3.2.1 Vibratory signal form

**Figure 4:**
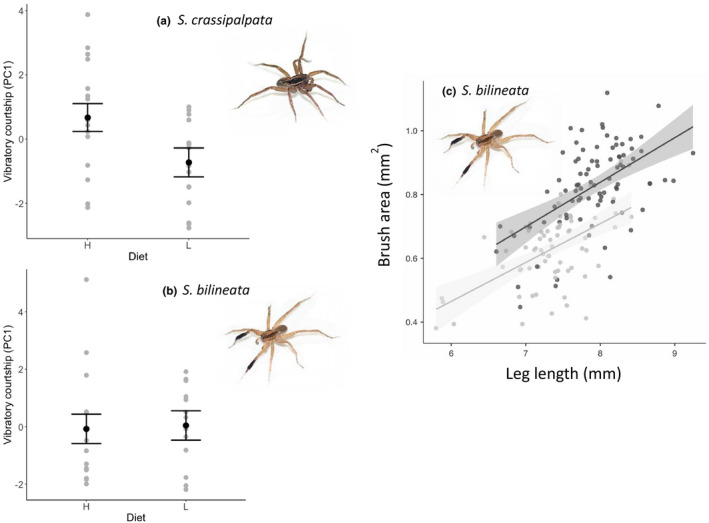
Vibratory signal form, as indicated by PC1 (# pulses, # trills, time spent courting), is diet‐dependent in *Schizocosa crassipalpata* (a), but not in *Schizocosa bilineata* (b). Error bars represent standard error. Brushes of HD (black line) male *S. bilineata* are larger relative to leg length than brushes of LD (gray line) male *S. bilineata* (c) (*p* < .0001) (HD: gray, LD: black; c). Shaded area represents the standard error

*Schizocosa crassipalpata*—The model that best predicted vibratory signaling (PC1) in *S. crassipalpata* (whole model: *R* ^2^ = 0.24, *F*_2,22_ = 3.56, *p* = .046) included diet (*b* = 0.70 ± 0.31, *F*_1,22_ = 4.93, *p* = .037) and relative leg length (*b* = −0.58 ± 0.33, *F*_1,22_ = 3.05, *p* = .095). High‐diet males produced more vibratory courtship (Figure 4a) and males with relatively longer legs tended to produce fewer vibratory signals, though leg length was not significant on its own.

*Schizocosa bilineata*—For *S. bilineata*, the model that best predicted the amount of vibratory courtship (PC1) males produced only included male CW (*R* ^2^ = 0.13, *F*_1,24_ = 3.47, *b* = 0.46 ± 0.25, *p* = .075). Larger males tended to produce more vibratory signaling, but again, this was not statistically significant.

In sum, vibratory signal form appears to be influenced by diet in *S. crassipalpata*, but not in *S. bilineata* (Figure 4).

#### 3.2.2 Visual signal form

*Schizocosa crassipalpata*—High‐diet (HD) males had significantly larger CW than low‐diet (LD) males (*t*_97_ = −4.62, *p* < .001; Table 1A). Relative leg length was greater in HD males than LD males (*t*_97_ = −2.77, *p* = .007; Table 1A;

). Relative mass was greater in HD males than in LD males (*t*_97_ = −4.00, *p* < .001; Table 1A).

**Table 1:** Effects of diet on adult male and female body measurements for (A) *Schizocosa crassipalpata* and (B) *Schizocosa bilineata* (X ± *SE*)

(A) *S. crassipalpata*								
	Male				Female			
	HD	LD	*t*	*p*	HD	LD	*t*	*p*
*N*	49	50			29	30		
Cephalothorax width (mm)	2.36 ± 0.12	2.23 ± 0.15	4.62	**<.001**	2.51 ± 0.13	2.36 ± 0.15	4.02	**<.001**
Relative leg length (mm)	0.10 ± 0.05	−0.010 ± 0.05	2.77	**.007**	−0.03 ± 0.06	0.03 ± 0.06	−0.74	.463
Relative mass (mg)	1.15 ± 0.40	−1.12 ± 0.40	4.00	**<.001**	2.14 ± 0.64	−2.07 ± 0.63	4.66	**<.001**

(B) *S. bilineata*								
	Male				Female			
	HD	LD	*t*	*p*	HD	LD	*t*	*p*
*N*	85	70			29	29		
Cephalothorax width (mm)	2.17 ± 0.12	1.98 ± 0.12	9.75	**<.001**	2.43 ± 0.11	2.22 ± 0.14	6.42	**<.001**
Relative leg length (mm)	−0.77x10−3 ± 0.04	0.94x10−3 ± 0.04	−0.03	.977	0.09 ± 0.09	−0.09 ± 0.09	1.54	.130
Relative mass (mg)	0.33 ± 0.23	−0.40 ± 0.25	2.18	**.031**	1.57 ± 0.97	−1.57 ± 0.97	2.29	**.026**
Relative brush area (mm^2^)	0.03 ± 0.01	−0.04 ± 0.01	4.38	**<.001**	NA	NA		

Diet and morphology did not predict leg wave height in *S. crassipalpata* (*t*_20_ = −0.05, *p* = .963). Leg wave duration depended on interactions between diet and both relative mass and wave height (whole model: *R* ^2^ = 0.65, *F*_5,16_ = 5.93, *p* = .003; diet: *b* = 0.08 ± 0.08, *p* = .318; relative mass: *b* = −0.32 ± 0.10, *p* = .006; wave height: *b* = 0.46 ± 0.25, *p* = .330; diet * relative mass: *b* = 0.34 ± 0.10, *p* = .004; diet * wave height: *b* = −0.10 ± 0.04, *p* = .017; Figure S2). In LD males, leg wave duration decreased with wave height and with increasing relative mass, while relative mass and wave height were not related to wave duration for HD males (Figure S2A). Essentially, LD males that were heavier for their size had a shorter wave duration, so legs moved faster for a given wave height.

*Schizocosa crassipalpata females*—High‐diet (HD) females had significantly larger cephalothorax widths than low‐diet (LD) females (*t*_57_ = −4.02, *p* < .001; Table 1A). We did not detect an effect of diet on relative leg length (*t*_57_ = 0.74, *p* = .463; Table 1A; Figure S2‐B). Relative body mass was higher in HD females than LD females (*t*_57_ = −4.66, *p* < .001; Table 1A).

*Schizocosa bilineata males*—HD males had significantly larger CW than LD males (*t*_153_ = −9.75, *p* < .001; Table 1B). Relative leg length did not significantly differ between the diet treatments (*t*_153_ = −0.03, *p* = .977; Table 1B). Relative body mass was greater in HD males than LD males (*t*_153_ = −2.18, *p* = .031; Table 1B). Relative brush area was greater in HD males than LD males (*t*_153_ = −4.38, *p* < .001; Table 1B; Figure 4c). We did not detect any effects of diet or morphology on leg wave height (diet: *t*_21_ = 0.47, *p* = .642) or leg wave duration (whole model: *R* ^2^ = 0.01, *F*_2,20_ = 0.02, *p* = .982; diet: *b* = 0.02 ± 0.09 *F*_1,20_ = 0.04, *p* = .852; wave height: *b* = 0.00 ± 0.05 *F*_1,20_ < 0.01, *p* = .998) (Figure S2‐B).

*Schizocosa bilineata females*—HD females had significantly larger CWs than LD females (*t*_56_ = −6.42, *p* < .001; Table 1B). Relative leg length was not significantly different between the diet treatments (*t*_56_ = −1.54, *p* = .130; Table 1B). Relative body mass was greater in HD females (*t*_56_ = −2.29, *p* = .026; Table 1B).

In sum, diet manipulations influenced body size (CW) and mass in females and males of both species (Table S2). Diet influenced relative leg length for *S. crassipalpata* males (Table 1) and brush area for *S. bilineata* males (Table 1; Figure 4c). In terms of the courtship signals we measured, we detected an influence of diet on the vibratory signal of *S. crassipalpata* (Figure 4a) and the visual wave duration of *S. crassipalpata's* foreleg tap (through a complex interaction with mass) (Figure S2). In *S. bilineata*, diet did not influence any dynamic signaling traits (Figure 4b; Figure S2).

## 4 OBJECTIVE III—SIGNAL FUNCTION ACROSS SIGNALING ENVIRONMENTS

In addition to the indirect approach of testing hypotheses of courtship signal function based upon signal form—for example, condition‐dependent signal expression might imply content‐based selection for indicator traits (for other examples, see Hebets & Papaj, 2005)—a more direct empirical approach can assess courtship signal function in terms of their importance with respect to mating success. Experiments that ablate, remove, or manipulate signal form and assess communication outcome (e.g., mating vs. no mating), for example, can directly test signal form‐to‐function relationships. Such approaches have been particularly useful in testing functional hypotheses of multimodal signaling (Balkenius & Dacke, 2010; Choi et al., 2019; Colyott et al., 2016), as scientists can determine the importance of modality‐specific signaling by artificially manipulating their presence/absence.

### 4.1 Methods

#### 4.1.1 Collection and maintenance

We used spiders from the same collecting trips as Objective II in this experiment. We housed spiders under the same conditions as described in Objective II, including the altered feeding regimes, or diet treatments.

#### 4.1.2 Experimental design

To determine the relative importance and function of vibratory and visual signaling in the courtship displays of *S. crassipalpata* and *S. bilineata,* we manipulated the signaling environment such that particular signaling channels (light and vibration) were isolated. We used a 2 × 2 design in which we controlled the presence/absence of light and vibration signal transmission independently (similar to Hebets, 2005, 2008; Hebets et al., 2013; Rundus et al., 2010, 2011).

Mating arenas across all signaling environments—light present/absent (L+/L−) and vibration present/absent (V+/V−) (all treatments: L+/V+; L+/V−; L−/V+; L−/V−)—consisted of bottomless circular plastic containers 12.5 cm in diameter (Pioneer Plastics Inc.) surrounded with white paper to control for any visual cues outside the arenas. We placed arenas on foam blocks to minimize ambient vibrational noise. Light present conditions were illuminated using two Virta Lite full‐spectrum 30‐watt fluorescent bulbs (Duro‐Test Lighting Inc.). We illuminated light absent conditions using only infrared illuminators (SuperCircuits) and observed interactions using Rigel 3200 night vision goggles (Rigel Optics Inc.). The infrared illuminator emitted light at a wavelength of ~850 nm. Electroretinogram recordings taken from a variety of wandering spiders, including wolf spiders, have provided no evidence that they are able to detect light at this wavelength (Barth et al., 1993; Devoe, 1972; Devoe et al., 1969).

Vibratory present conditions consisted of filter paper (Schleicher and Schuell) lining the arena floor. We ran vibration absent conditions in a bottomless arena on granite. Previous work using a laser Doppler vibrometer to record male *Schizocosa* wolf spiders producing vibrational signals have shown that granite is not effective at transmitting a male spider's signals (Elias & Mason, 2014; Rundus et al., 2010). We ran groups of four L+ trials back‐to‐back with groups of four L‐trials. Within each light treatment, we ran two replicates of V+ and two replicates of V−.

Approximately 24 hr before the start of trials, we fed females one size‐matched cricket to minimize the potential for precopulatory cannibalism. We placed females in their respective arenas at least 1 hr prior to the start of the trial to acclimate and deposit silk cues. These cues alone are enough to elicit courtship behaviors from both male *Schizocosa* wolf spiders (Roberts & Uetz, 2005; Vaccaro et al., 2010; MB personal observation). Trials began when we introduced males into the arena. We allowed each pair to interact for up to 30 min. We live‐scored trials for male leg waves (and subsequently used this to calculate courtship rate), copulation presence/absence, and time to copulation. We used leg waves as our proxy of courtship rate so that our scoring could be comparable to other *Schizocosa* courtship rate scores to date (e.g., Rosenthal & Hebets, 2012, 2015; Rundus et al., 2011; Shamble et al., 2009).

We ran a total of 131 *S. crassipalpata* females (66 HD, 65 LD) and 131 *S. crassipalpata* males (62 HD, 69 LD) through mating trials. Females ranged between 11 and 19 days postmaturation with a mean age of 14.9 days postmaturation. We ran a total of 193 female *S. bilineata* (104 HD and 89 LD) and 193 male *S. bilineata* (105 HD and 88 LD) through mating trials. Females ranged in age from 11 to 16 days postmaturation with a mean of 14.12 days postmaturation. We used each spider for each species only once.

*Statistical Analyses*—For each species, we first compared female and male age across diet treatments using a nonparametric Wilcoxon test. Because we found female age to be diet‐dependent in both *S. crassipalpata* and *S. bilineata* (see Objective III Results), we included female diet in subsequent analyses. Additionally, because male courtship signals vary with male diet differently across the species (see Objective II Results), and because male age varied with diet in *S bilineata* (see Objective III Results), we include male diet in all subsequent analyses. We also included diet * environment interactions (vibratory and visual).

To determine whether our diet treatments and/or signaling environment manipulations influenced copulation success, we used a logistic regression with the independent variables of female diet, male diet, light environment, vibratory environment, and interactions between light environment * vibratory environment, male diet * light environment, and male diet * vibratory environment. The response variable was copulation (yes/no).

Next, using only data on pairs that copulated, we ran a least‐squares regression model with the same independent variables to test whether the time to copulation was dependent on diet and/or signaling environments. Since there were no matings in some of the environment treatments (i.e., L−/V−), we were unable to include interaction terms in these models. Furthermore, because time to copulation was not normally distributed, our response variable for these models was the log‐transformed time (s) to copulate. For all analyses, we used only trials in which males engaged in courtship for at least 10 s. This resulted in the removal of six males that did not court (*n* = 6) and one that courted for less than 6 s (*n* = 1) in *S. crassipalpata*, and for eleven males that did not court (*n* = 11) in *S. bilineata*.

We calculated the courtship rate for each species as the total number of leg waves divided by the total time spent courting. Similar measures of courtship rate have been strongly associated with mating success in other *Schizocosa* species (Hebets et al., 2011; Rosenthal et al., 2018; Shamble et al., 2009). To determine whether courtship rate might help explain our observed patterns of copulation, we compared male courtship rate across diet and signaling environments. Specifically, we used a standard least‐squares regression model with the same independent variables as in our copulation model (female diet, male diet, light environment, vibratory environment, light environment * vibratory environment, male diet * vibratory environment, male diet * light environment). Because courtship rate was not normally distributed, we used a cubed root transform of courtship rate as our response. We ran all analyses with the statistical software JMP.

### 4.2 Results

*Schizocosa crassipalpata—*There was a significant difference in the average female age, as measured from the ultimate molt to the time used in a trial, between diet treatments at the time of trials (Wilcoxon's signed‐rank test, Z = −5.64, *df* = 1, *p* < .001). HD females averaged 15.71 days postmaturation, while LD females averaged 14.08 days postmaturation. Males ranged between 12 and 37 days postmaturation and averaged 17.76 days postmaturation. There was no difference in the average age between male diet treatments (Wilcoxon's signed‐rank test, *Z* = 1.34, *df* = 1, *p* = .18). Diet‐dependent age differences reflect diet's influence on maturation time, with HD females maturing slightly earlier than LD females and thus achieving a greater “age” at the time of trials.

Since we had a limited number of individuals to use across our experimental treatments for *S. crassipalpata*, we stopped conducting trials in the vibratory absent environments after the first 48 mate choice trials (12 trials run in each of the four environmental treatments). At this point, only a single pair had copulated in the vibration absent environments (1 L+/V−; 0 L−/V−), while 17 pairs had copulated in the vibration present treatments (10 L+/V+; 7 L−/V+). Our rationale for continuing with the vibration present environments was to ensure that we would have a large enough sample size to detect signal interactions should they be present (e.g., Stafstrom & Hebets, 2013; Wilgers & Hebets, 2012).

**Figure 5:**
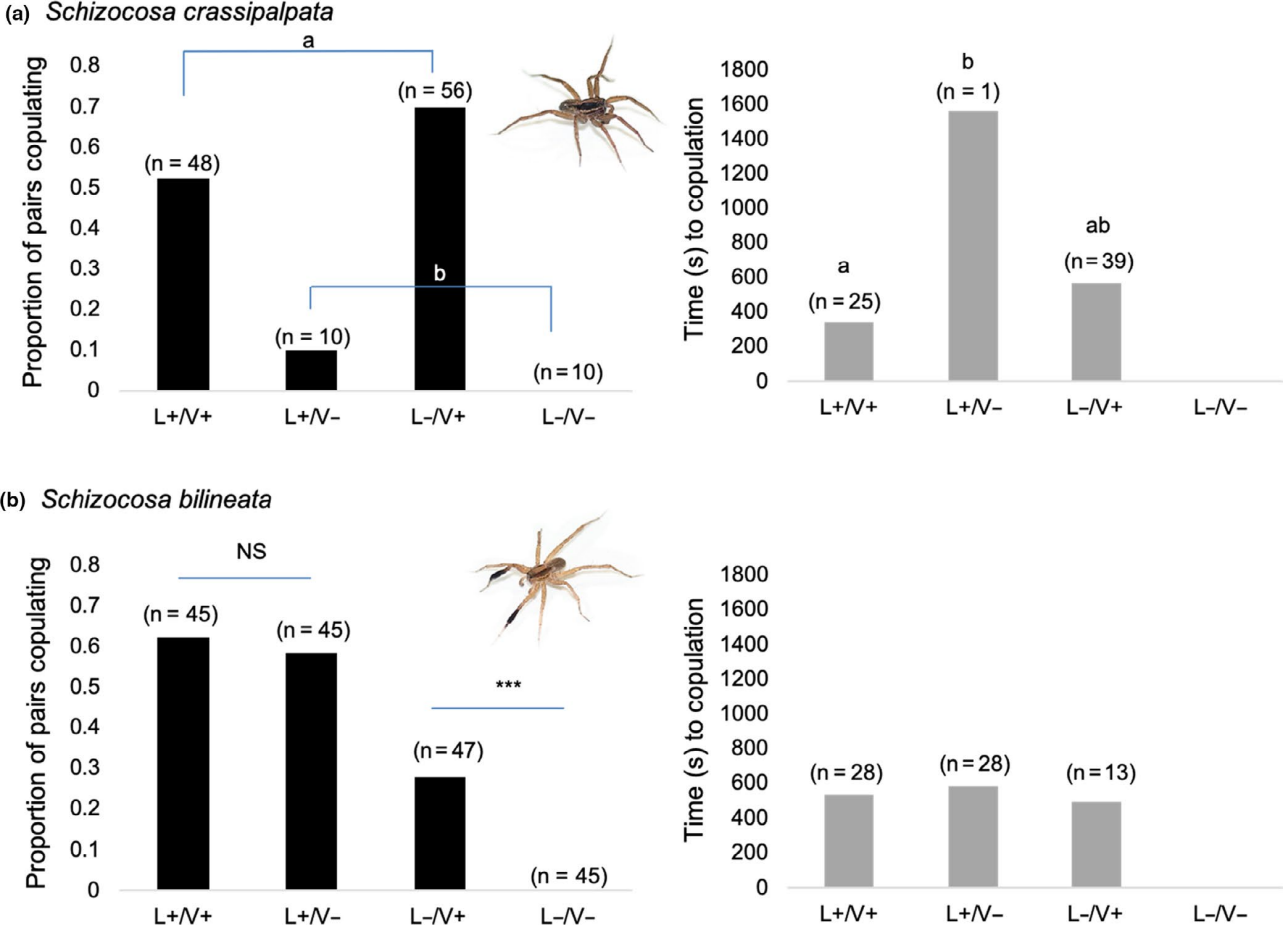
*Schizocosa crassipalpata* pairs were more likely to mate in the presence (V+) versus absence (V−) of vibration (a—left panel). Copulations took longer in the light than in the dark, though sample sizes were negligible in the light (a—right panel). *Schizocosa bilineata* pairs were more likely to mate in the light (L+) versus the dark (L−) and the presence of vibratory signaling only mattered in the dark (b—left panel). There was no influence of the signaling environment on time to mating for *S. bilineata* (b—right panel)

Courtship and Copulation in *S. crassipalpata*—Mating success in *S. crassipalpata* was influenced by the vibratory environment but not by diet (female or male) or the light environment (overall model: *df* = 7, chi‐square = 32.92, *p* < .001; Table 2A). Pairs were more likely to mate in the presence, versus absence, of vibratory signaling (likelihood‐ratio test: chi‐square = 28.6, *p* < .001; Figure 5a—left panel). There were no significant interactions (Table 2A). There was also no difference in the distribution of male diet treatments across the signaling environments (chi‐square = 1.62; *p* = .65).

**Table 2:** Variables predictive of copulation success (Left Panel, ChiSquare statistics) and latency to copulation (Right panel, *F* ratio statistics) for (A) *Schizocosa crassipalpata* and (B) *Schizocosa bilineata.*

(A) *S. crassipalpata*		Copulation Success		Latency to Copulation	
Variable	*df*	ChiSquare	*p*‐value	*F* ratio	*p*‐value
Female diet	1	0.97	.32	1.42	.24
Male diet	1	1.74	.19	1.28	.26
Light Environ.	1	1.09	.30	7.76	**.007**
Vibratory Environ.	1	28.60	**<.0001**	5.36	**.02**
Light*Vib. Environ.	1	2.77	.10		
Male diet*Vib. Environ.	1	1.86	.17		
Male diet*Light Environ.	1	0.53	.46		

(B) *S. bilineata*		Copulation Success	Latency to Copulation
Variable	*df*	ChiSquare	*p*‐value	*F* ratio	*p*‐value
Female diet	1	0.16	.69	0.74	.39
Male diet	1	0.03	.87	4.42	**.04**
Light Environ.	1	59.72	**<.0001**	0.15	.7
Vibratory Environ.	1	12.97	**.0003**		
Light*Vib. Environ.	1	13.60	**.0002**		
Male diet*Vib. Environ.	1	0.97	.33		
Male diet*Light Environ.	1	0.05	.82		

Note: Bold indicates significance.

Because there was only one mating in the V− treatment, we were unable to include an interaction term in the model for time to copulation. The time to copulation was influenced by the light environment and the vibratory environment (overall model: *df* = 4, *F* = 3.71, *p* = .009; Table 2A). When we conducted a Tukey–Kramer HSD comparison of means, we found that the time to mating in the L+/V− treatment was significantly longer than in the L+/V+ (Figure 5a—right panel). We note, however, that we only had one mating in the L+/V− environment.

**Figure 6:**
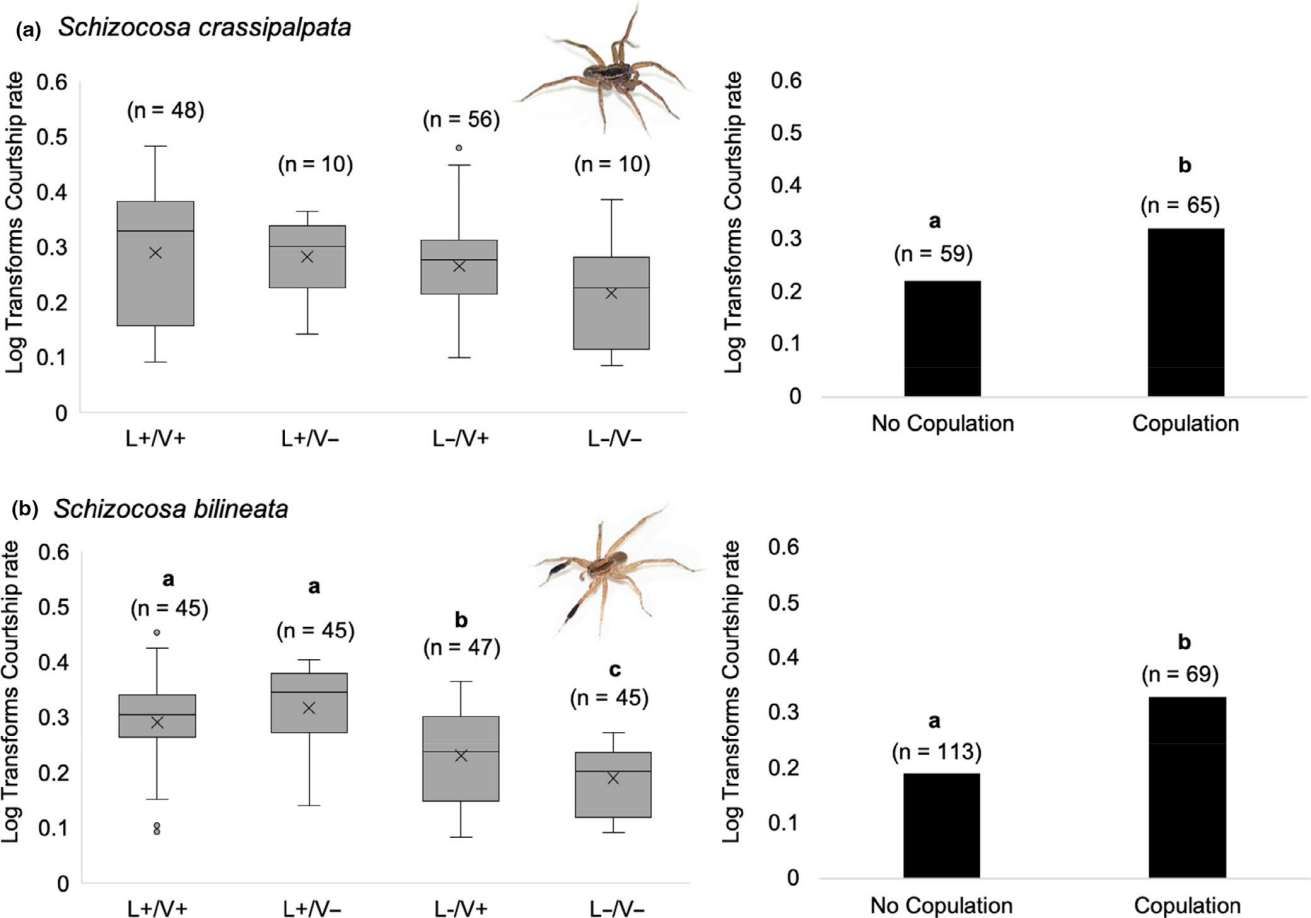
The courtship rate of *Schizocosa crassipalpata* males was independent of the signaling environment (a—left panel), but males that copulated showed a higher courtship rate than those that did not (a—right panel). In contrast, *Schizocosa bilineata* males courted fastest in the light conditions (L+), regardless of the vibratory environment. They showed a slower courtship rate in the dark, but with vibration present and courted the slowest in the dark with no vibration present (b—left panel). Like *S. crassipalpata*, males that copulated showed higher courtship rates

Male *S. crassipalpata* courtship rate was not influenced by diet (female or male), the signaling environment, or any interaction (overall model: *df* = 7, *F* = 0.88, *p* = .52; Figure 6a—left panel). However, males that copulated had a higher courtship rate than males that did not copulate (ANOVA: *df* = 1, *F* = 43.00, *p* < .0001; Figure 6a—right panel).

*Schizocosa bilineata*—Similar to *S. crassipalpata*, there was a difference in the mean age between female diet treatments (Wilcoxon's signed‐rank test, Z = −2.811, *df* = 1, *p* < .005). HD females averaged 14.29 days postmaturation, while LD females averaged 13.92 days postmaturation. Males ranged in age from 10 to 30 days postmaturation with an average of 19.66 days. There was a difference in the average age between male diet treatments (Wilcoxon's signed‐rank test, *Z* = −6.1, *df* = 1, *p* < .001). HD males averaged 21.75 days postmaturation, while LD males averaged 17.13 days postmaturation.

Courtship and Copulation in *S. bilineata*—Copulation success in *S. bilineata* was influenced by an interaction between the light environment * vibratory environment, but not by diet (female or male) (overall model: *df* = 7, chi‐square = 68.51, *p* < .0001; Table 2B). Both light and vibratory environments individually also showed significance in the model (Table 2B). In the light present, there was no difference in copulation success across the vibratory environment (likelihood‐ratio test: chi‐square = 0, *p* = 1), but in the light absent, pairs were more likely to copulate in the presence, versus absence, of a vibratory signal (likelihood‐ratio test: chi‐square = 19.52, *p* < .0001; Figure 5b—left panel). None of our variables were good predictors of the time to copulation for *S. bilineata* (overall model: ANOVA, *df* = 4, *F* = 1.06, *p* = .39; Figure 5b—right panel). There was also no difference in the distribution of male diet treatments across the signaling environments (chi‐square = 1.26; *p* = .74).

Male *S. bilineata* courtship rate was influenced by an interaction between light * vibratory environment, but not by female or male diet (overall model: *df* = 7, *F* = 7.93, *p* < .0001; female diet: *F* = 0.03, *p* = .86; male diet: *F* = 0.03, *p* = .86; light environment, *F* = 43.55, *p* < .001; vibratory environment: *F* = 0.93, *p* = .34; light * vibratory environment: *F* = 7.38, *p* = .007; male diet * light: *F* = 1.07, *p* = .30; male diet * vibration: *F* = 2.63, *p* = .11; Figure 6b—left panel). A Tukey–Kramer HSD test revealed that males courted at a higher rate in the light present. In addition, in the light present there was no effect of the vibratory environment, but in the light absent, courtship rate was lower in the absence versus presence of a vibration (Figure 6b—left panel). Furthermore, similar to in *S. crassipalpata*, males that copulated had a higher courtship rate than those that did not copulate (ANOVA: *df* = 1, *F* = 239.01, *p* < .0001; Figure 6b—right panel).

## 5 OBJECTIVE IV—ACTIVITY CYCLES

Given the divergence in modality‐specific courtship signal reliance we found between *S. crassipalpata* and *S. bilineata*, we next wanted to test the hypothesis that the species also diverged in their temporal activity patterns. Specifically, given the diet dependence and reliance on vibratory signaling, we predicted that *S. crassipalpata* would be more nocturnally active. In contrast, we expected the visually ornamented *S. bilineata*, with condition‐dependent brushes and a high mating success in the light, to be more diurnally active.

### 5.1 Methods

We collected *S. crassipalpata* from Bath Nature Preserve in Summit County, OH (41.1766°N, 81.6480°W), from 29 to 30 April 2019 and *S. bilineata* from The Ohio State University campus at Newark, Licking County, OH (40.0753°N, 82.4425°W), from 1 to 2 April 2018. To determine the natural activity cycle of each species, we monitored individual spider activity for 16 mature females of each species and 15 mature male *S. crassipalpata* and 16 mature male *S. bilineata*. We monitored locomotion for 5 days with a 12‐hr:12‐hr light:dark cycle that matched the light cycle in the laboratory. Lights went on at 08:30 hr and off at 20:30 hr.

We placed each spider in a 25 mm diameter × 125 mm length glass tube, with half of a wet 10 × 38 mm dental cotton roll inside each end of the tube to provide moisture. We inserted the tubes into a locomotor activity monitor (model LAM) from Trikinetics, Inc., which houses four rows of eight tubes. The activity monitor recorded movement as the number of times spiders interrupted one of three infrared beams that pass through the center of each tube. The monitor counted movement in 5‐min bins. There is no existing evidence to suggest that wolf spiders are capable of detecting infrared, and thus, these beams should not impact movement (Barth et al., 1993; Devoe, 1972; Devoe et al., 1969).

We covered the tubes with masking tape to limit visual interactions between spiders. We left a single gap that allowed the infrared beams to pass through the midline of each tube. Light was provided with a full‐spectrum compact fluorescent light bulb (NATURESSUNLITE 30 W; Naturallighting.com, Dickinson, Texas). To minimize disturbance and the influence of other light sources, we placed the entire setup in an isolated room accessible only through another dark room. No one entered throughout the duration of the experiment, and thus, there should have been no external visual stimuli beyond the experimental setup. We placed spiders in the activity monitor for 6 days and measured activity for days 2–6, thus allowing the spiders 1 day of acclimation.

For each individual, we calculated the proportion of total activity that took place during the light phase. These proportions were logit‐transformed for analyses. We used *t* tests to determine whether the mean proportion for each sex of each species was significantly different from 0.5 (representing equal activity during the light and dark). We used an ANOVA with species, sex, and their interaction to determine whether the proportion of diurnal activity differed across the groups. Data were back‐transformed for presentation here.

### 5.2 Results

Both *S. crassipalpata* and *S. bilineata* females and males demonstrated similar movement patterns, with a trend toward a higher magnitude of the increase in activity following the dark phase in *S. crassipalpata* females and males (Figure S3). For both species, activity was high during the early daylight hours and tapered off just before the lights went out. Following the lights going out, there was an increase in movement, with all groups of spiders again tapering off their activity toward the end of the dark period. For mature female and mature male *S. crassipalpata* and female *S. bilineata*, activity was significantly higher when the lights were on, while activity was similar across the light cycle for female *S. bilineata* (Table 3). We did not, however, detect significant differences in the proportion of diurnal activity between species and sexes (ANOVA: *F*_3,59_ = 0.27, *p* = .844).

**Table 3:** Proportion of total activity that took place during the photophase (light) and whether it was significantly different from 0.5 for each sex of each species

Species	Sex	*N*	Mean	95% CI	Range	*t*	*p*
*Schizocosa crassipalpata*	M	15	0.590	0.535–0.644	0.419–0.747	3.45	**0.004**
	F	16	0.570	0.512–0.626	0.345–0.758	2.57	**0.021**
*Schizocosa bilineata*	M	16	0.591	0.535–0.644	0.418–0.760	3.43	**0.004**
	F	16	0.563	0.493–0.630	0.308–0.751	1.92	0.074

## 6 DISCUSSION

*Schizocosa crassipalpata* and *S. bilineata* are closely related wolf spiders that show different patterns of sexual dimorphism and differ in the diet dependence of, and reliance on, vibratory and visual courtship signals. Our results first corroborate their phylogenetic status, as we found support for their sister‐species relationship following a robust molecular analysis. Our findings place *S. duplex* as their closest relative and suggest that the sexually dimorphic brushes of *S. bilineata* are independently derived. In the monomorphic species, *S. crassipalpata*, we found that male vibratory courtship signaling was diet‐dependent and crucial for mating success. Relative foreleg length was also diet‐dependent in male, but not female, *S. crassipalpata*, suggesting sexually selected leg length in males. In contrast, the sexually dimorphic *S. bilineata* males showed diet‐dependent brush area (but not leg length nor vibratory signaling) and a strong reliance on visual signaling for mating success. Although *S. bilineata* pairs mated most in the presence of visual signaling, vibratory signaling was important in the dark. The apparent increased reliance on visual signaling in *S. bilineata*, in addition to field experiences in which *S. bilineata* was consistently more easily collected during the day (Pers. Obs), led us to hypothesize that *S. bilineata* might be more day‐active—that is, that the two species have diverged in temporal activity patterns as well. Our activity monitoring data, however, found no major differences in activity patterns between the sexes or the species. The potential for temporal partitioning, however, still exists, as our experimental design was unable to detect courtship‐specific activity or potential crepuscular activity. Following from our results, we hypothesize that visual signaling in *S. bilineata* evolved in response to competition for signaling space and potentially facilitated species divergence.

Our phylogeny, based on >500 nuclear loci, resembles phylogenies based on mitochondrial DNA and morphology (Fowler‐Finn et al., 2015; Hebets et al., 2013; Stratton, 2005), but with strong support at all nodes. Previous research using only morphological traits initially hypothesized a sister‐species relationship between *S. crassipalpata* and *S. bilineata* (Stratton, 2005). Our results support this hypothesis and indicate that the single‐hooded epigynum is a synapomorphy for *S. bilineata* and *S. crassipalpata* (Dondale & Redner, 1978). Preliminary analyses of a more inclusive data set including data from all North American *Schizocosa* further corroborate the sister‐species relationship between *S. crassipalpata* and *S. bilineata* (Starrett et al., unpublished data). We also find support for *S. crassipalpata* + *S. bilineata* as a sister group to *S. duplex*. Like *S. crassipalpata*, *S. duplex* lacks tibial ornamentation (Hebets & Uetz, 2000; Stratton, 2005) and all three species rely on stridulation for courtship (Stratton, 2005). Similar to previous hypotheses (Stratton, 2005), our results suggest an independent and secondary gain of tibial bristles (or brushes) in *S. bilineata*. More comprehensive species sampling for subgenomic level sequence data and a formal analysis of ornamentation are now needed to more rigorously test this hypothesis.

In addition to the expected diet‐dependent morphological traits in females and males of both species (e.g., CW, mass), we found diet‐dependent relative leg length in male *S. crassipalpata*, but not in females nor either sex of *S. bilineata*. We also found diet‐dependent brush area in male *S. bilineata*. Such patterns of diet dependence are consistent with sexually selected traits developing a higher degree of condition dependence than nonsexually selected traits (Bonduriansky, 2007; Cotton et al., 2004a; Rowe & Houle, 1996). For both species, males incorporate foreleg waving into their courtship displays, consistent with a putative role for forelegs in sexually selecting signaling.

The foreleg wave duration in *S. crassipalpata* was diet‐dependent through a complex interaction with weight and leg wave height. In particular, LD males that were heavier had a shorter duration leg wave (i.e., faster speed) with higher leg waves. Given the importance of courtship rate on mating success seen in this and other *Schizocosa* species (*S. ocreata*: Stoffer et al., 2016, Delaney et al., 2007, *S. retrorsa*: Hebets et al., 1996, *S. stridulans*: Hebets et al., 2011, Rosenthal & Hebets, 2015, *S. uetzi*: Shamble et al., 2009), it is possible that LD males in better condition were “making up for their small size” with faster than expected signaling. Such behavioral adjustments might allow males to signal greater performance capacity (Byers et al., 2010). A similar pattern was found in male *S. stridulans*, where males with less foreleg ornamentation had a mating advantage if they courted at higher rates (Hebets et al., 2011). The function of leg waving in *S. crassipalpata*, however, remains unclear, as vibratory signaling (not visual) played a predominant role in the mating success of this species. Nonetheless, it is possible that visual leg waving interacts with other environmental factors and/or other display traits in a complex manner that we were unable to detect (e.g., Rosenthal & Hebets, 2012), or that it reflects the production of signals in an alternate signaling modality—for example, near‐field sound (Choi et al., 2019; Rundus et al., 2010). This latter explanation seems unlikely though, given that we observed no mating in the L−/V− signaling environments.

An alternative, or additional, potential function of diet‐dependent foreleg length in *S. crassipalpata* may be to help males mount females by reducing the likelihood of sexual cannibalism. Indeed, sexual dimorphism in leg length appears to play a role in reducing sexual cannibalism in other spider taxa—for example, the nursery web spider *Pisaurina mira* (Anderson & Hebets, 2016). In *S. crassipalpata,* no males were cannibalized during any of our mating trials, but this may be a direct result of male foreleg dimorphism. The ratio of leg length to body size (as measured by CW) is greater for males than females in both species (data not shown). Our data cannot address any of these hypotheses directly. We now need future research to examine potential reproductive functions of the observed diet‐dependent foreleg length in *S. crassipalpata*.

The diet dependence of brush area in *S. bilineata* is unsurprising, as our results are consistent with multiple studies on other *Schizocosa* demonstrating the diet‐dependent expression of sexual ornaments (Hebets et al., 2008, 2011; Rosenthal & Hebets, 2012; Rundus et al., 2011; Shamble et al., 2009; Uetz et al., 2002). In *S. ocreata*, brush size is additionally known to be an honest indicator of immune function (Gilbert et al., 2016; Gilbert & Uetz, 2016). Also similar to previous research, and despite the diet dependence of brush area in *S. bilineata*, we observed no influence of male diet (either on its own or through interactions with the signaling environment) on mating success. Previous studies across ornamented *Schizocosa* species similarly observe that diet‐dependent ornamentation does not necessarily influence male mating success (*S. stridulans*: Rosenthal & Hebets, 2015, *S. uetzi*: Shamble et al., 2009, *S. floridana*: Rosenthal & Hebets, 2012, Rosenthal et al., 2018, *S. ocreata*: Scheffer et al., 1996), though female choice for larger brushes has been suggested in *S. ocreata* through assays of female receptivity (reviewed in Uetz et al., 2017). As mentioned by previous authors, diet‐dependent trait expression does not necessarily reflect selection for indicator traits, nor does it equate to adaptive mate choice (Patricelli et al., 2019).

Similar to the diet‐dependent brush area of *S. bilineata*, vibratory signaling was diet‐dependent in *S. crassipalpata*, yet male mating success was independent of diet, or any interaction between diet and the signaling environment. Direct individual‐level variation in male vibratory courtship songs and its relationship with female mate choice has only been examined in *S. ocreata* thus far (Gibson & Uetz, 2012). Female *S. floridana*, however, do appear to use vibratory signaling as an indicator of male condition when it is the only signaling modality available. Using a similar experimental design as the present study, Rundus et al. (2011) found that female *S. floridana* mated more often with high‐diet males in dark, but not light, conditions (Rundus et al., 2011). Future research exploring the natural relationship between song characteristics and mate choice as well as artificial song manipulations and playbacks to females is now necessary to determine the extent to which female choice might influence the evolution and function of *S. crassipalpata* courtship song.

Finally, in relation to the importance of vibratory signaling in *S crassipalpata*, we note that we presume that our observed differences in mating success across environments result from female choice and not plastic male behavior. Though some males may have been less motivated to court in the light (5/99 males did not court and all were in light present environments), the signaling environment did not influence the rate at which male *S. crassipalpata* courted.

As the presumed ancestral signaling modality in *Schizocosa*, vibratory signaling retains a role in the mating success of both focal species, though in a different manner. In *S. crassipalpata*, successful mating was dependent on the presence of vibratory signaling, whereas for *S. bilineata*, vibratory signaling was only important for mating success in the dark. Vibratory signaling has been shown to be the dominant signaling modality across numerous previously studied *Schizocosa* species (*S. duplex*, *S. rovneri*, *S. saltatrix*: Hebets et al., 2013), and even in the more distantly related *Rabidosa rabida* wolf spiders (Wilgers & Hebets, 2012). Our results corroborate earlier suggestions regarding the universal importance and ancestry of vibratory signaling in *Schizocosa*.

*Schizocosa bilineata* is one of the first species for which there appears a strong reliance on visual signaling. Over 50% of pairs mated across the light environments, regardless of the presence of vibratory signaling. In contrast, zero pairs mated in the absence of visual and vibratory signaling and less than 30% mated in the presence of vibration only. *Schizocosa bilineata* is one of only a handful of species in the genus that possess conspicuous foreleg brushes (Stratton, 2005). For the only other conspicuously brushed species that have been studied—*S. ocreata* and *S. crassipes*—isolated visual and vibratory signals are equally important for eliciting female receptivity (*S. ocreata*: Uetz et al., 2009, Kozak & Uetz, 2019) or for facilitating mating success (*S. crassipes*: Stafstrom & Hebets, 2013). But responses to multimodal signaling are higher than to either isolated signaling modality, suggesting an additive or interactive influence of multimodal signaling in these species. In the absence of vibratory signaling, there is mixed evidence for a female preference for increased ornamentation—that is, data suggest preference for increased ornamentation (*S. crassipes*: Hebets & Uetz, 2000, *S. ocreata*: Scheffer et al., 1996); data suggest no preference for ornamentation (*S. ocreata*: McClintock & Uetz, 1996, *S. crassipes*: Stafstrom & Hebets, 2013). In the presence of full multimodal courtship, however, numerous studies in *S. ocreata* (McClintock & Uetz, 1996; Persons & Uetz, 2005; Uetz et al., 2017) (but see Scheffer et al., 1996) and *S. crassipes* (Stafstrom & Hebets, 2013) suggest that males with larger brushes are preferred.

Due to the presence of conspicuous foreleg ornamentation in *S. bilineata*, it is tempting to interpret our results of higher mating success in light solely through the lens of female choice for elaborate condition‐dependent ornamentation. The absence of diet‐dependent mate choice, however (i.e., no effect of diet on mating success), makes this explanation of female choice for indicator ornaments unsatisfactory. Nonetheless, brushes may still play a role in mate choice in *S. bilineata*. For example, the presence or size of male brushes may increase the efficacy of leg waving by increasing its detectability or discriminability against complex backgrounds or in different contexts (Guilford & Dawkins, 1991; Hasson, 1991). This hypothesis has been previously suggested for other species of wolf spider where foreleg ornamentation was not found to be under direct sexual selection (Shamble et al., 2009). Visual displays that increase the signal efficacy in complex, or "noisy," backgrounds exist (Ord et al., 2007) and could provide an important mechanism driving the evolution of male brushes in *S. bilineata*. The addition of an alternate signaling modality (e.g. visual) might be particularly important in environments where closely related species overlap in habitat and in ancestral signaling modalities.

Finally, the synchrony of vibratory and visual signaling in *S. bilineata* is notable, especially given that female *S. ocreata* respond more to synchronous versus asynchronous multimodal signaling (Kozak & Uetz, 2016). The potential for intersignal interactions or synergy between vibratory and visual signaling in *S. bilineata*, especially as it relates to brush size, will be an important area for future studies.

Although the signaling environment did not influence the likelihood that male *S. bilineata* courted, the courtship rate of male *S. bilineata* was environment‐dependent (unlike the environment‐independent courtship rate of *S. crassipalpata*). In particular, we observed an identical pattern in courtship rate as we did mating success—males in the light environments (L+/V+; L+/V−) courted at the highest rate, followed by the L−/V+, and the lowest courtship rate was observed in the L−/V−. For both *S. crassipalpata* and *S. bilineata*, males that acquired a mating courted at a higher rate than those that did not.

Courtship rate has been the most consistent predictor of mating success across *Schizocosa* (*S. crassipalpata* and *S. bilineata*: present study, *S. ocreata*: Delaney et al., 2007, *S. retrorsa*: Hebets et al., 1996, *S. uetzi*: Shamble et al., 2009, *S. stridulans*: Hebets et al., 2011, Rosenthal & Hebets, 2015, *S. retrorsa*: Rundus et al., 2010, *S. ocreata*: Stoffer et al., 2016). Female preference for high male courtship rate has been documented across additional taxa such as fiddler crabs (Murai & Backwell, 2005), crickets (Tregenza et al., 2006), and damselfish (Knapp & Kovach, 1991). This pattern is consistent with the hypothesis that female mate choice is based upon male motor performance (Byers et al., 2010). Past studies, however, have demonstrated that males will increase their courtship rate if they receive positive feedback from receptive females (Sullivan‐Beckers & Hebets, 2011). Thus, it is unclear whether males that court at a higher rate are more attractive to females, or whether males that are more attractive ultimately court at a higher rate because of female feedback, or some combination of both. Unfortunately, the cause and effect of courtship rate and mate choice are impossible to disentangle in our experiments.

In conclusion, instead of differential (micro)habitat use driving divergence in *S. crassipalpata* and *S bilineata*, we propose that shared habitat and competition for signaling space initiated signal divergence, specifically selection for visual signaling. In a multispecies assemblage consisting of multiple ground‐dwelling arthropods that rely upon vibratory signaling, one potential avenue for reducing signal interference, or increasing signal to noise ratios, is to add an additional signaling modality. In particular, we propose that *S. bilineata* added visual signaling to avoid heterospecific interference from *S. crassipalpata*. Alternatively, it is entirely possible that these species partition the microhabitat in a more subtle way than we are aware to avoid competition, or that courtship signaling may have diverged in allopatry and extant sympatric populations reflect secondary contact.

Furthermore, though there is overlap in the temporal patterning of vibratory courtship displays between *S. crassipalpata* and *S. bilineata*, there are clear differences as well. Whether these differences are sufficient for species recognition remains an open question. In other closely related *Schizocosa* species—*S. uetzi* and *S. stridulans*—vibratory signals are sufficient to maintain species‐specific matings (Hebets, 2007). In field crickets, *Teleogryllus oceanicus* and *T. commodus*, females also show species‐specific selectivity for different song features, enabling accurate species recognition (Bailey et al., 2017). The extent to which *S. crassipalpata* and *S. bilineata* females can distinguish conspecifics based solely on vibratory courtship signals remains unknown, as does the potential for hybridization. Additionally, the extent to which vibratory signals might differ in their degree of overlap in sympatry versus allopatric populations is unknown. Such findings might support the hypothesis that vibratory signal interference has led to the evolution of a secondary signaling modality—visual signaling—in *S. bilineata*.

In recent years, communication system elements (e.g., display components, the signaling environment, receiver biology) have repeatedly been shown to interact in a way that influences communication function, revealing that a focused approach on one system element might not provide accurate information on overall system form and function (Hebets et al., 2016). There is an increased awareness of, and attention to, the numerous distinct sources of selection that can influence the evolutionary patterns of communication displays (reviewed in Bradbury & Vehrencamp, 2011; Hebets & McGinley, 2019; Patricelli & Hebets, 2016). Given the (a) diversity of multimodal courtship displays among *Schizocosa*, especially between closely related species; (b) evidence for the microhabitat (i.e., substrate type) influencing vibratory signal transmission; (c) evidence for different patterns of diet‐dependent morphology and multimodal signaling reliance across species; and (d) a lack of clear patterns with respect to female choice for secondary sexual ornaments; *Schizocosa* wolf spiders provide an ideal system for unraveling the relative roles and importance of different drivers of signal divergence/convergence. Future studies that add data for new species in a phylogenetic context and incorporate a systems approach—an approach that explored form‐to‐function relationships across environments and contexts (Hebets et al., 2016)—will surely help with this effort.

## CONFLICT OF INTEREST

None declared.

## AUTHOR CONTRIBUTIONS

**Eileen A. Hebets:** Conceptualization (lead); funding acquisition (lead); project administration (lead); resources (lead); supervision (lead); writing–original draft (equal); writing–review and editing (lead). **Mitch Bern:** Data curation (lead); formal analysis (equal); investigation (lead); methodology (lead); writing–original draft (equal); writing–review and editing (supporting). **Rowan H. McGinley:** Formal analysis (equal); writing–review and editing (supporting). **Andrew J. Roberts:** Investigation (supporting); writing–review and editing (supporting). **Arik Kershenbaum:** Formal analysis (supporting); writing–review and editing (supporting). **James Starrett:** Formal analysis (equal); investigation (equal); writing–review and editing (supporting). **Jason E. Bond:** Data curation (equal); formal analysis (equal); funding acquisition (equal); project administration (supporting); supervision (supporting); writing–review and editing (supporting).

## Supporting information

Video S1

Video S2

Appendix S1

## Data Availability

Data for Objective I: https://doi.org/10.25338/B8933F. Data for Objectives II–IV: https://doi.org/10.5061/dryad.dncjsxkxw.
