# Supplementary material for: Sister species diverge in modality‐specific courtship signal form and function
Source: Ecol Evol. 2020 Dec 30;11(2):852–71. doi: 10.1002/ece3.7089 (PMC7820158; doi:10.1002/ece3.7089)
Supplement: Supplementary file 3 — Appendix S1 [file ECE3-11-852-s003.docx]

**Appendix Tables and Figures**

**Table S1.** Collection information for spiders used in phylogenetic analyses of anchored enrichment sequences.


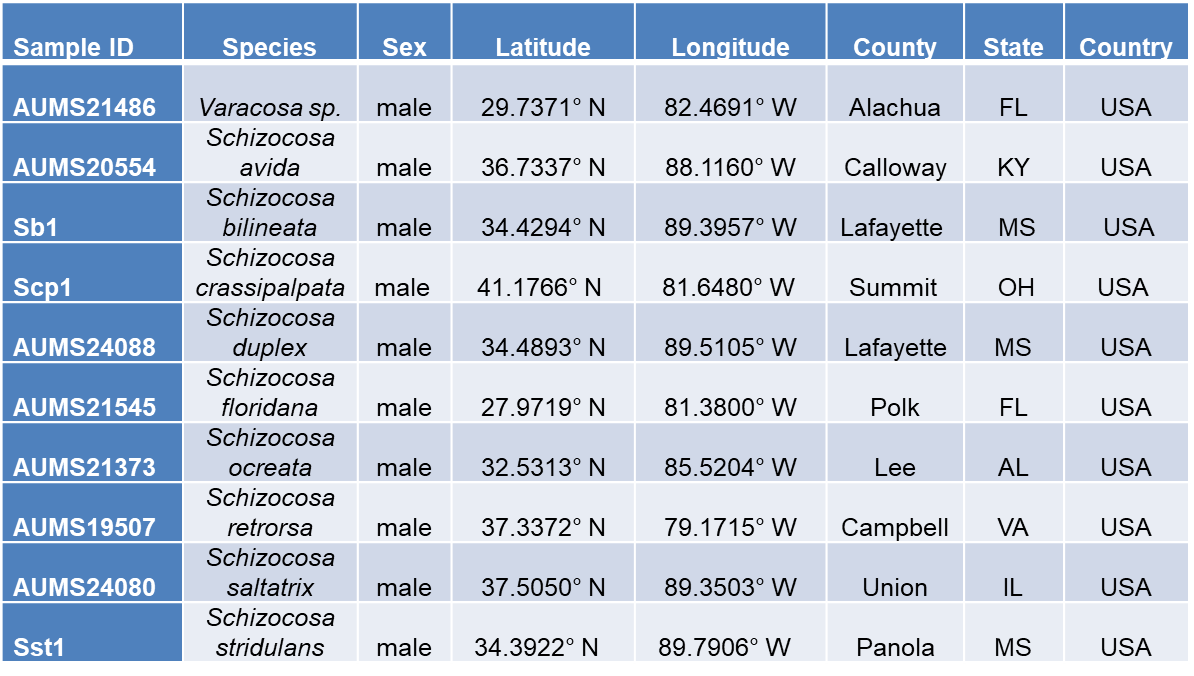


**Table S2.** Effects of diet on adult male and female body measurements for **(A)** *S. crassipalpata* and **(B)** *S. bilineata* (X±SD).


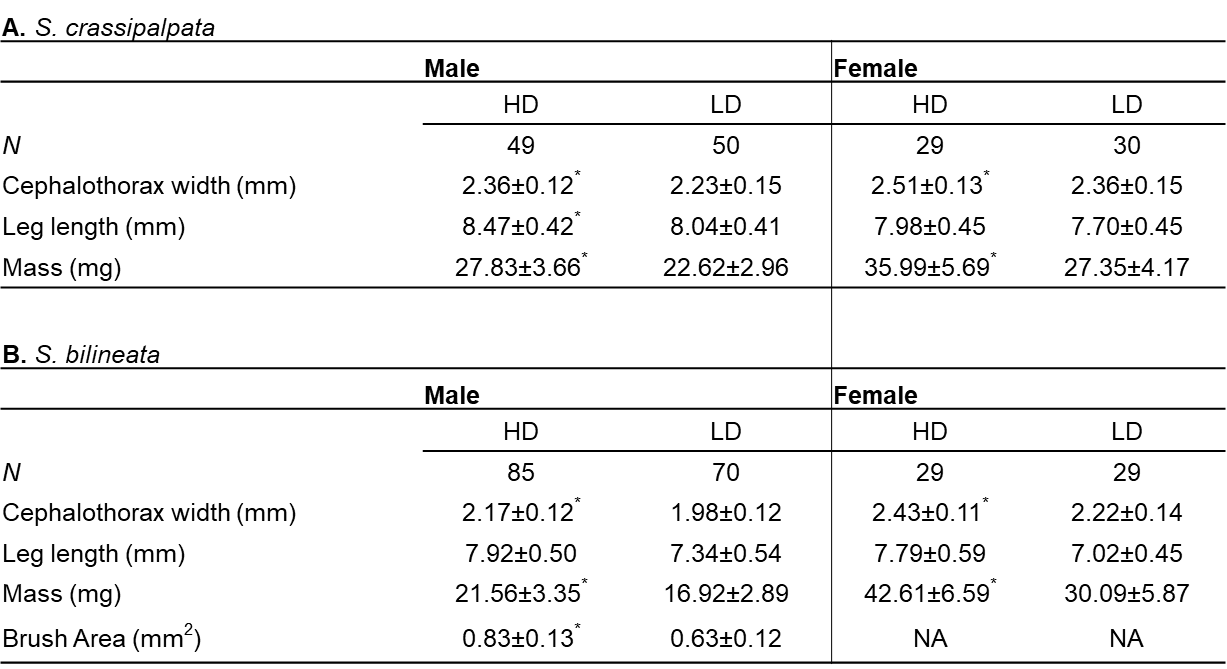


^*^Indicates that HD individuals had significantly larger values than LD individuals

**Figure S1**


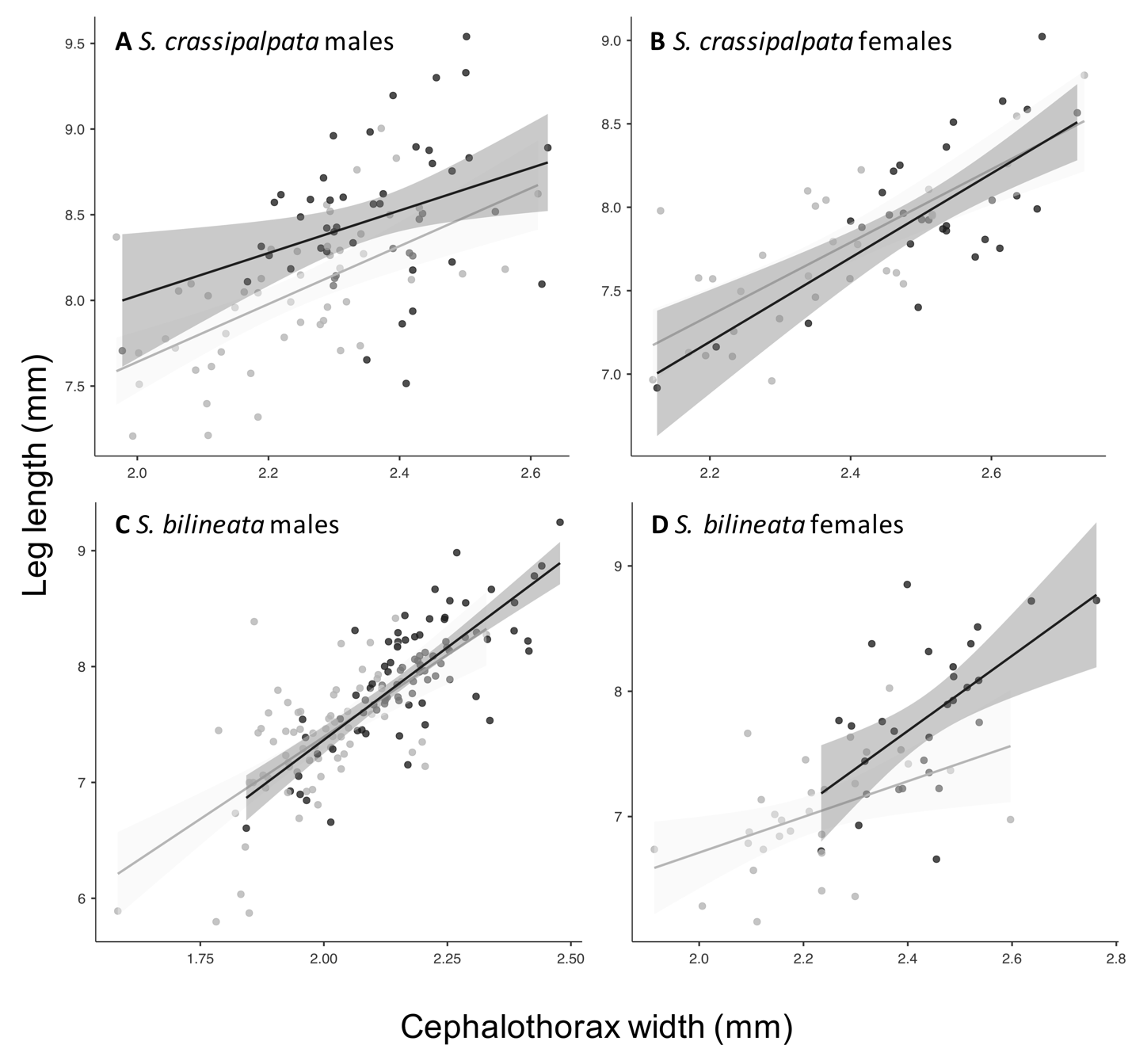


**Figure S1.** Leg length increases as body size (cephalothorax width) increases for *S. crassipalpata* males (**A**) and females (**B**) and *S. bilineata* males (**C**) and females (**D**) (HD: black line, LD: grey line). Leg length is relatively longer in HD than LD *S. crassipalpata* males.

**Figure S2**

**
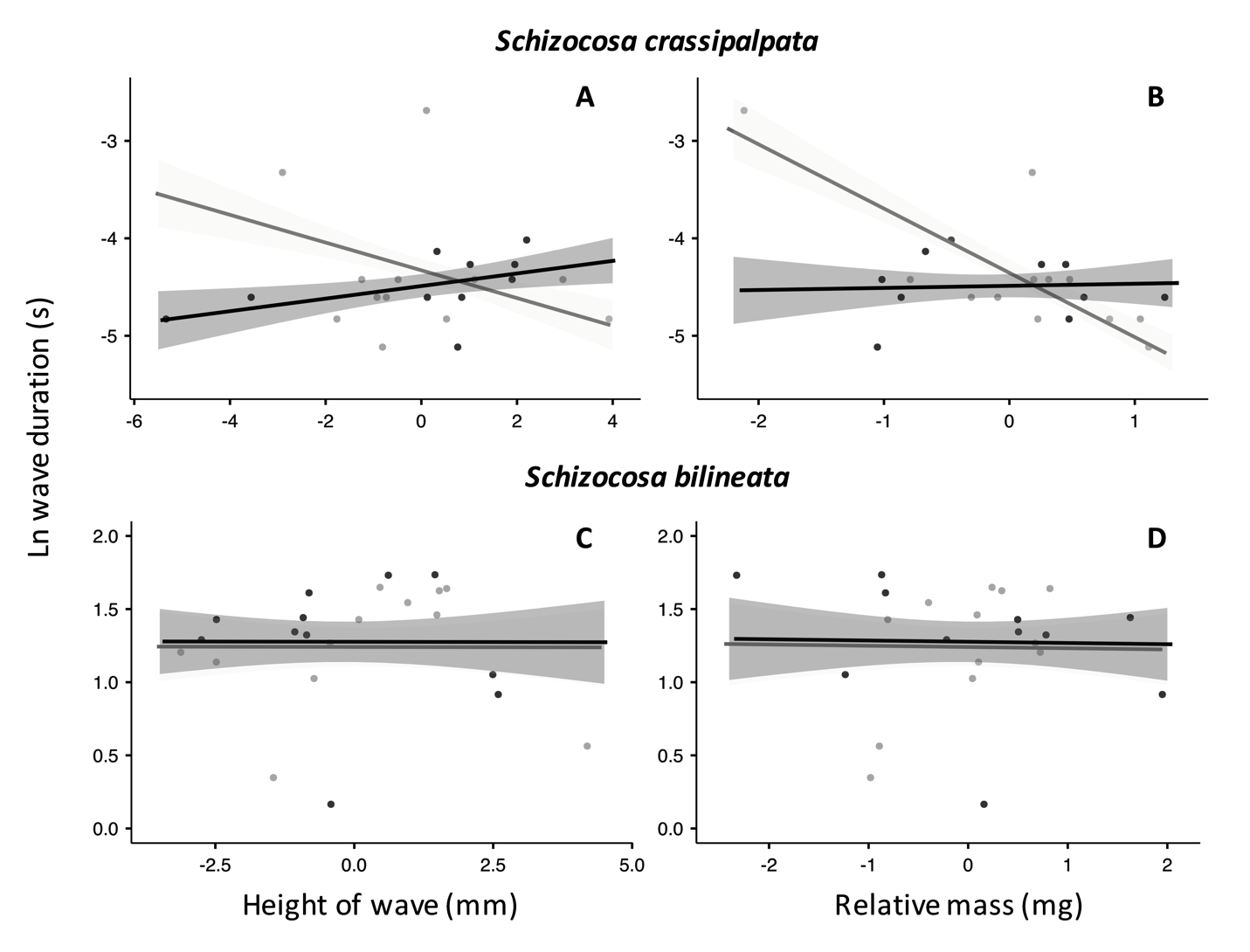
**

**Figure S2** Low Diet (LD: grey line) *Schizocosa crassipalpata* males (**Top Row**) displayed shorter leg wave duration (i.e. faster leg waving) when they had larger mass and higher waves. No such effect of diet, mass, or leg wave height was seen in *S. bilineata* (**Bottom Row**).

**Figure S3**


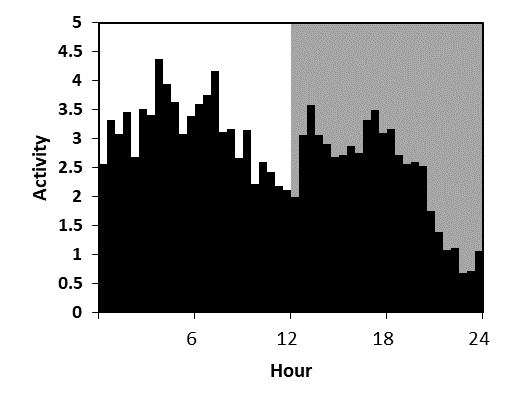

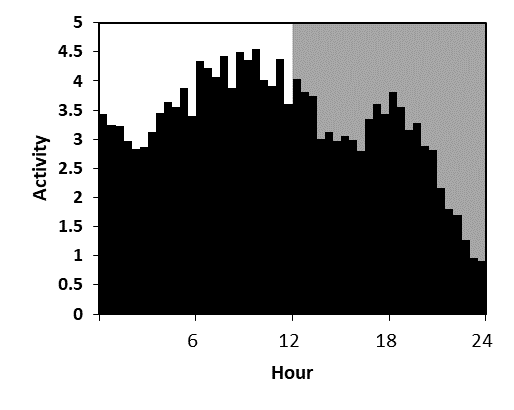


**B** *S. crassipalpata* females

**D** *S. bilineata* females


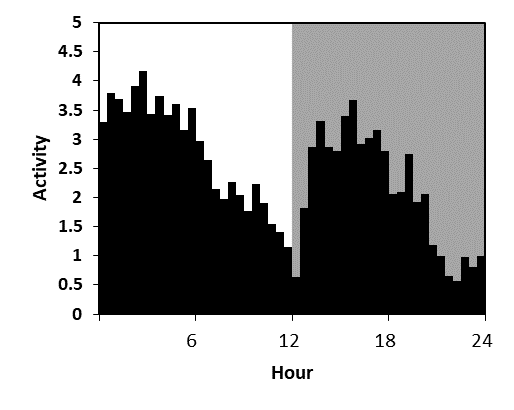

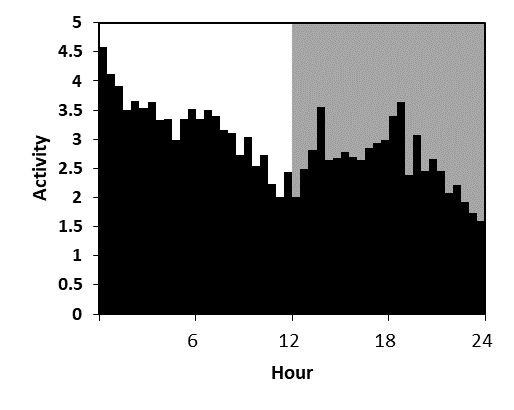


**A** *S. crassipalpata* males

**C** *S. bilineata* males

**Figure S3.** Both *S. crassipalpata* males (**A**) and females (**B**) as well as *S. bilineata* males (**C**) and females (**D**) show activity during both light and dark periods. The rise in activity following the dark period is qualitatively sharper for *S. crassipalpata* as compared to *S. bilineata*.
